# The “C” in Complexity: A Case Series on Navigating Fan’s Classification of C-shaped Canals

**DOI:** 10.7759/cureus.98699

**Published:** 2025-12-08

**Authors:** Aabha B Rawal, Dhaval V Desai, Riddhi V Doshi, Hetvi K Bhuva

**Affiliations:** 1 Conservative Dentistry and Endodontics, Government Dental College and Hospital, Jamnagar, IND; 2 Conservative Dentistry and Endodontics, Faculty of Dental Science, Dharmsinh Desai University, Nadiad, IND; 3 Conservative Dentistry and Endodontics, Private Practice, Ahmedabad, IND; 4 Public Health, Reflections Dental Care, Brooklyn Park, USA

**Keywords:** c-shaped canal configuration, endodontic treatment, fan's classification, root canal anatomy, themoplasticized obturation

## Abstract

C-shaped canal anatomy is most commonly found in mandibular second molars. The complex anatomy can be challenging to the clinician during negotiation, debridement, and obturation. The fan-shaped regions, transverse anastomoses, fins, and webs of the C-shaped anatomy can be identified and treated with the help of radiographic and clinical diagnosis. Adequate knowledge of the root canal configuration is essential to achieve success in endodontic therapy. This case series highlights the management of four different cases of C-shaped canal configurations categorized on the basis of Fan's Classification. Despite the significant challenges, a positive outcome was achieved in all the cases through specific protocols and modified obturation techniques.

## Introduction

A good understanding of the root canal anatomy is essential for the success of root canal therapy. The root canal anatomy of the mandibular second molars varies greatly. The root canal system's "C" configuration is the most prevalent among them, which was first reported by Cooke and Cox in 1979 [1]. Manning [2] proposed that the main reason for the C canal configuration might be the inability of Hertwig's epithelial root sheath to fuse onto the buccal or lingual root surface. Instead of having multiple distinct orifices, this form of canal arrangement has a single ribbon-shaped orifice that resembles a 180° arc that begins at the mesiolingual line angle and arcs across the buccal to the distal side. The two main variations are (a) a single, ribbon-like, C-shaped canal that extends from the aperture to the apex and (b) three or more separate canals that emerge beneath the C-shaped orifice [3].

With a prevalence ranging from 2.7% to 44.5%, the C-shaped variations were most frequently found in mandibular second molars [4]. Additionally, maxillary first molars (0.12%), maxillary third molars (4.7%), mandibular third molars (3.5-4%), and mandibular second premolars (1%) have also been observed to have "C" shaped canals [5]. It is not associated with gender, age, or tooth position. However, ethnic predilection is noted, with the highest incidence reported in East Asian population groups such as Chinese (29.7%) and Koreans (31.3%-45.5%) [6].

Various classifications have been published to make it easier to diagnose and treat variations of C-shaped canals. Melton [7] divided them into three groups based on their cross-section: continuous C-shaped, semicolon-shaped, and two or more distinct canals. Fan et al. [8] later modified this classification, as the main drawback of Melton's Classification was that there was no clear description of the difference between categories C2 and C3. Four distinct C-shaped canal configurations in mandibular second molars, classified from C-1 to C-4 of Fan's Anatomic classification, are presented in this case series.

## Case presentation

**Figure 1:**
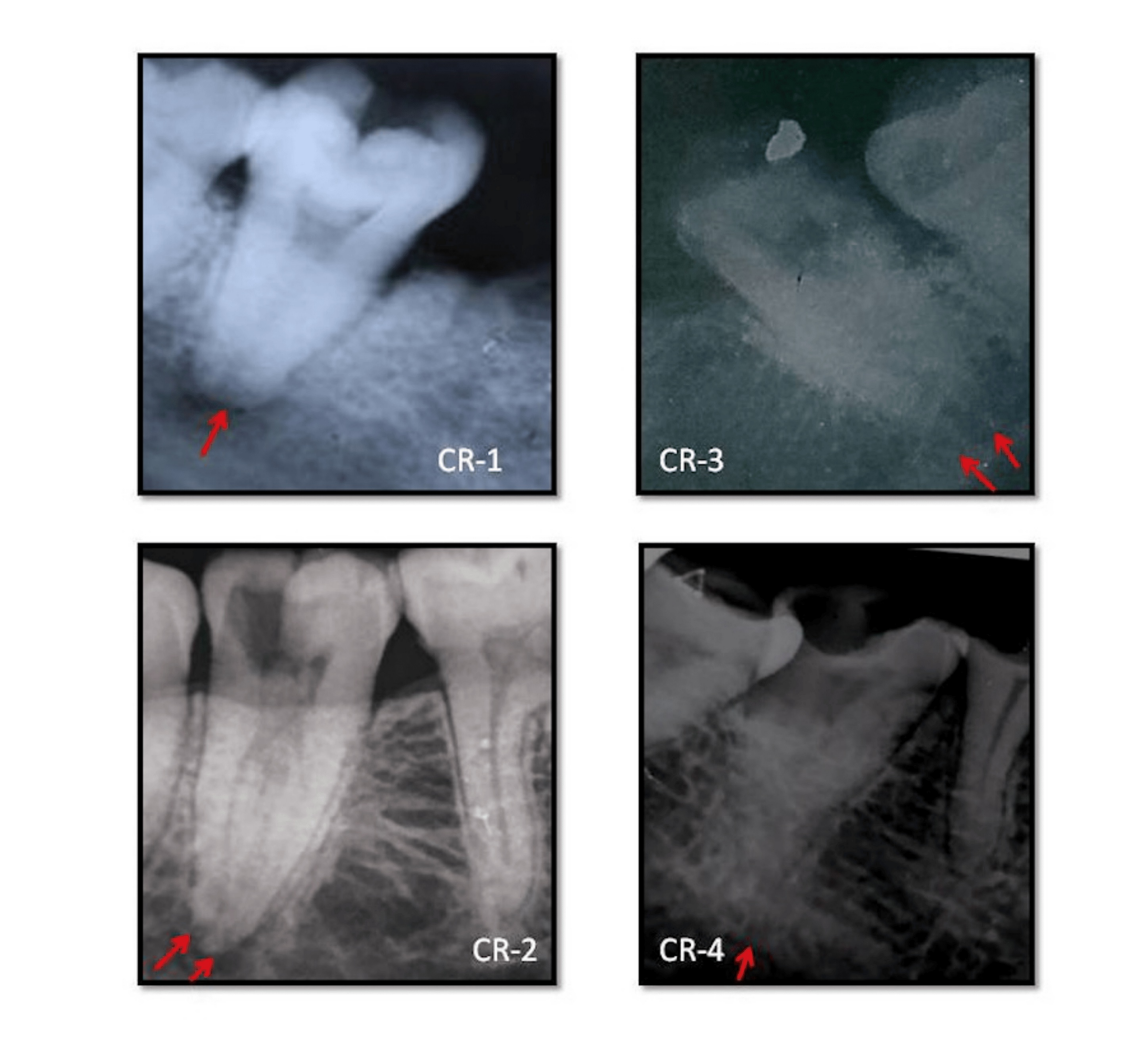
Preoperative radiographs CR-1: A single square shaped root; CR-2: Fused mesial and distal roots; CR-3: Fused mesial and distal roots; CR-4: A single conical root. CR: case report.

Based on the patient's history of pain, clinical examination, results of pulp sensibility test - electric pulp test (Analog Vitality Pulp Tester, Parkell Inc., Edgewood, NY, USA), and radiographic examination, the diagnosis of each case has been summarized in Table 1. As shown in Figure 1, preoperative radiographs revealed conical roots in case report (CR)-1 and CR-4, and fused roots in CR-2 and CR-3.

**Table 1:** Case history and diagnosis CR: case report. PDL: periodontal ligament.

Case Report	Age / Gender	Clinical features	Tenderness on percussion	Pulp Sensibility Test	Radiographic findings	Diagnosis
CR-1	38/Female	A history of dull, persistent pain several months back that subsided after a few weeks.	Negative	No response	Occlusal radiolucency approaching the pulp, with widening of the PDL space and loss of lamina dura	Pulpal necrosis with asymptomatic apical periodontitis
CR-2	32/Female	Dull aching, continuous pain, moderate in intensity and intermittent in nature.	Positive	Delayed response	Occlusal radiolucency approaching the pulp, with widening of the PDL space and loss of lamina dura	Symptomatic irreversible pulpitis with symptomatic apical periodontitis.
CR-3	45/Male	Spontaneous, dull aching, aggravated on chewing, and relieved on taking medications	Positive	Delayed response	Disto-occlusal radiolucency approaching the pulp	Symptomatic irreversible pulpitis with symptomatic apical periodontitis
CR-4	55/Male	Pain on mastication	Negative	No response	Occlusal radiolucency approaching the pulp, with widening of the PDL space and loss of lamina dura	Pulpal necrosis with symptomatic apical periodontitis

**Figure 2:**
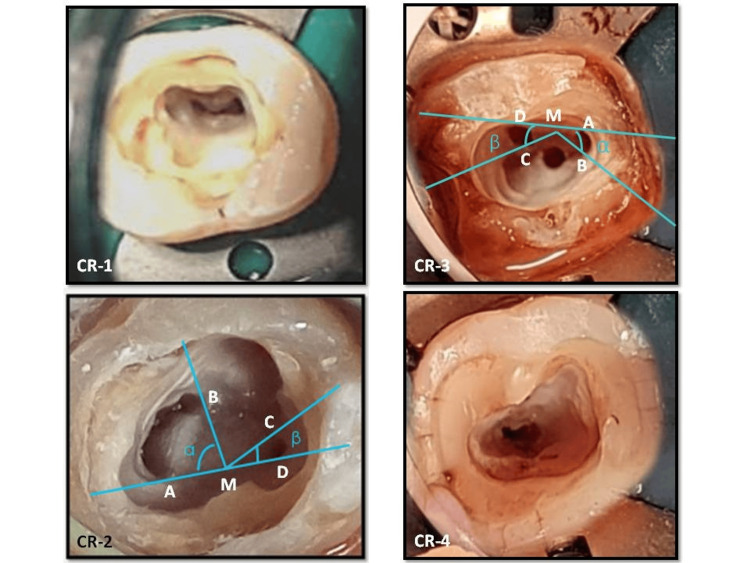
Application of Fan's (modified anatomic) classification to clinical appearance of pulp chamber floor CR-1: A continuous uninterrupted "C"; CR-2: semicolon shaped with angle α greater than 60°; CR-3: three separate orifices with both the angles α and β were less than 60°; CR-4: single oval canal. (Measurements of angles α and β:  A and B, ends of one canal cross-section; C and D, ends of the other canal cross-section; M, middle point of line AD; α,angle between line AM and line BM; β, angle between line CM and line DM).

Based on the diagnosis, root canal treatment was planned for the teeth over two visits. After administration of local anaesthesia, traditional endodontic access opening was done with an Endo Access Bur (Dentsply Tulsa, Tulsa, Oklahoma, USA). On clinical inspection of the pulp chamber floor, as shown in Figure 2, all the cases were classified as given in Table 2.

**Table 2:** Categorization of the C-shaped anatomy CR: case report.

Classification based on:	Preoperative Radiograph	After Access opening	After Access opening	WL Radiograph	Obturation Radiograph
Classification type:	Fan's Radiographic classification 2004 [9]	Melton's Anatomic classification 1991 [7]	Fan's Modified anatomic classification 2004 [8]	Fan's Radiographic Classification 2004 [9] ​​​​​​	Fan's Radiographic Classification 2008 [10]
CR-1	Type I - A conical or square root that is divided into distal and mesial sections by a hazy, radiolucent longitudinal line. The mesial and distal canals were present, and they merged into one before leaving at the apical foramen.	Category 1 - An uninterrupted C-shaped outline is defined by a continuous C-shaped canal that extends from the pulp chamber to the apex.	Category 1 - the shape is an uninterrupted “C” with no separation or division	Type I - A conical or square root that is divided into distal and mesial sections by a hazy, radiolucent longitudinal line. The mesial and distal canals were present, and they merged into one before leaving at the apical foramen.	Type I (merging type)-canals merge into one major canal in the apical
CR-2	Type II - A conical or square root that is divided into distal and mesial sections by a hazy, radiolucent longitudinal line. Both mesial and distal canals were present, and they both seemed to proceed independently to the apex.	Category 2 - the semicolon-shaped orifice in which dentine divides one mesial distinct canal from a major C-shaped canal.	Category 2 - the canal shape resembles a semicolon resulting from a discontinuity of the “C” outline, but either angle α or β should not be less than 60°	Type II - A conical or square root that is divided into distal and mesial sections by a hazy, radiolucent longitudinal line. Both mesial and distal canals were present, and they both seemed to proceed independently to the apex.	Type III (asymmetrical type)-separate mesial and distal canals, both canals appear to be asymmetrical in their size and continue as separate canals on their own pathway to the apex, in which the distal canal appears much wider than the mesial canal
CR-3	Type II - A conical or square root that is divided into distal and mesial sections by a hazy, radiolucent longitudinal line. Both mesial and distal canals were present, and they both seemed to proceed independently to the apex.	Category 3 - those with two or more distinct, independent canals. Subdivision I- C-shaped orifice in the coronal third that divides into two or more separate canals that merge apically.	Category 3 - two or three separate canals, and both angles, α and β, were less than 60°	Type I - A conical or square root that is divided into distal and mesial sections by a hazy, radiolucent longitudinal line. The mesial and distal canals were present, and they merged into one before leaving at the apical foramen.	Type I (merging type)-canals merge into one major canal in the apical
CR-4	-	-	Category 4 - only one round or oval canal in that cross-section	-	-

Case report one

First Appointment

**Figure 3:**
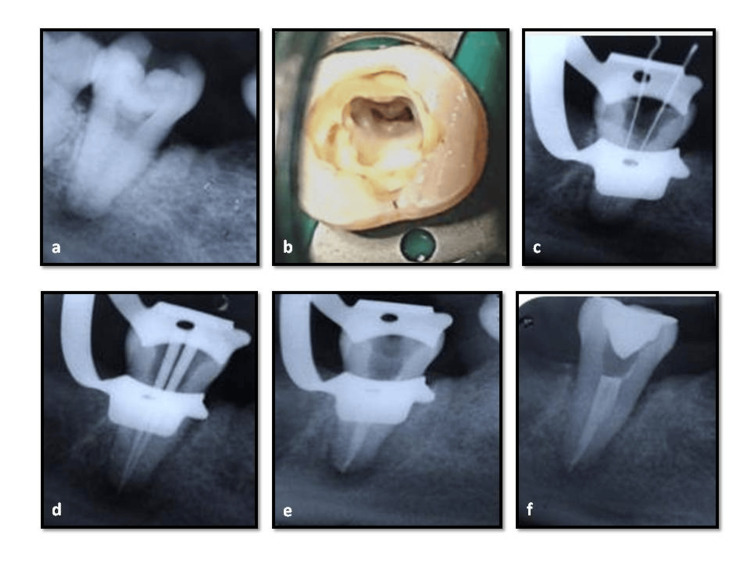
Case report-1 a) Preoperative radiograph shows square-shaped, fused roots; b)access opening, a single, large, c-shaped canal configuration with two orifices interconnected with a isthmus; c)working length radiograph shows narrow isthmus between the mesial and distal canals; d) master cone radiograph; e), f) post-obturation radiographs.

The preoperative diagnostic radiograph (Figure 3a) revealed a square root outline. A single, large C-shaped canal was found at the centre of the floor of the pulpal chamber, following the access opening (Figure 3b). Two orifices at both ends of the C were negotiated, which further merged into a single C-shaped canal. An apex locator (Root ZX II, Morita, Tokyo, Japan) was used to determine working length, and radiographs were used to corroborate the results(Figure 3c). For cleaning and shaping, the circumferential filing technique was used using ISO 2% taper hand files up to size 25 (Mani Inc., Tochigi-Ken, Japan). Additionally, 3% sodium hypochlorite (Vishal Dental Products, Mumbai, India) was utilized as an irrigant in between instrumentation, and cleaning and shaping were completed with appropriate ProTaper Gold rotary files (Denstply Sirona, Switzerland) up to F3. 5ml of 17% EDTA (RC-Prep, Premier Dental, Medical Product Laboratories, Inc., Philadelphia, USA) with 3ml of saline (Aculife Healthcare Pvt. Ltd., Gujarat, India) for one minute was used for the removal of the smear layer. This was followed by 3ml of 3% sodium hypochlorite for one minute per canal, 3ml of saline for one minute per canal, and 3ml of 17% EDTA for 1 minute per canal, coupled with sonic agitation of the irrigants for one minute per canal using EndoActivator (Dentsply Maillefer, USA). Normal saline was used for the canal's final rinse. As an intracanal medicament, calcium hydroxide paste (Prevest Denpro Calcigel, India) was placed into canals for one week, and closed with a temporary dressing. 

Second Appointment

The intracanal dressing was removed from the canals at the subsequent visit, and root canal irrigants were used to clean each canal. Obturation was scheduled since, upon clinical examination, the patient was asymptomatic. Master-cone selection was done and verified by a radiograph (Figure 3d). The canal was dried using absorbent points, and the apical third was obturated using the sectional technique. This was followed by backfill with thermoplasticized gutta-percha using the Eighteenth fast fill (Orikam, Changzhou Sifary Medical Technology Co. Ltd., China) and AH Plus resin sealer (Dentsply Maillefer, USA) (Figure 3e, 3f).

Case report two

First Appointment

**Figure 4:**
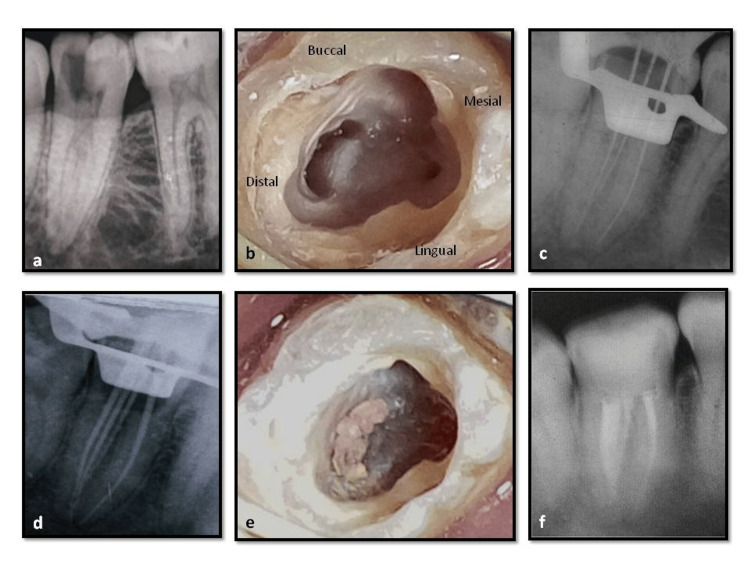
Case report-2 a) Preoperative radiograph, fused mesial and distal roots; b) access opening, mesiobuccal canal orifice with a groove extending continuously to the distal canal with a distinct mesioligual canal, typical semicolon shaped configuration; c) working length radiograph; inserted instruments give the impression of passing through the furcation area; d) master cone radiograph, mesiobuccal and distal canals coverging in the apical third and a separate mesiolingual canal; e) postobturation clinical photograph; f) postoperative radiograph.

The preoperative radiograph (Figure 4a) exhibited fused mesial and distal roots, leading to modified access cavity preparation. A mesiobuccal orifice and a groove that extended continuously down the buccal wall to the distal canal orifice were seen on the pulpal floor with a distinct, circular mesiolingual canal orifice indicating a typical semi-colon-shaped C-shaped canal configuration. The mesiolingual canal continued as a separate canal from the orifice to the apex (Figure 4b). Three orifices were recognized during pulp chamber exploration and were negotiated using a #10 K file (Mani Inc., Tochigi-Ken, Japan). Working length was determined using an apex locator and confirmed using an intraoral periapical radiograph (Figure 4c). To guarantee optimum pulp tissue removal, biomechanical preparation was carried out in the mesiobuccal, distobuccal, and distolingual canals using NeoEndo S rotary files (Orikam, Changzhou Sifary Medical Technology Co. Ltd., China) up to the 25/.04 master apical file, along with 3% sodium hypochlorite and 17% EDTA. This was followed by circumferential filing with 2% taper hand K-files. The mesiolingual canal was also prepared up to 25/.04 master apical file along with 3% sodium hypochlorite in between instrumentation. Irrigation protocol was the same as mentioned in case report one. Calcium hydroxide paste (Prevest Denpro Calcigel, India) was placed in the canals as an intracanal medicament for one week. After that, a temporary dressing was given, and the patient was recalled for the next appointment.

Second Appointment

As the patient was asymptomatic, 25/.04 master cones (Dia-Dent Gutta-percha points, Korea) were coated with Grossman's sealer (Dentsply Ind.e Com. Ltda., Brazil) and inserted in the mesiobuccal, distobuccal, and distolingual canals (Figure 4d). Using size 25 and 20 finger spreaders and 20/.02 and 15/.02 taper accessory gutta-percha cones, cold lateral compaction was carried out starting from the mesial portion of the canal to the distolingual portion of the canal, then continuing around the canal periphery until the spreader could only penetrate 2-3 mm into the canal. After the entire canal was filled, the excess gutta-percha was removed with a hot instrument to just below the cementoenamel junction. The obturated material was then vertically condensed using a heated hand plugger. Later, another master gutta-percha cone 25/.04 taper was coated with Grossman’s sealer, inserted to working length into the separate mesiolingual canal, and seared off at the canal orifice(Figure 4e). Post-endodontic composite resin (Charisma, Kulzer, Germany) restoration was done, and a postoperative radiograph was taken (Figure 4f).

Case report three

**Figure 5:**
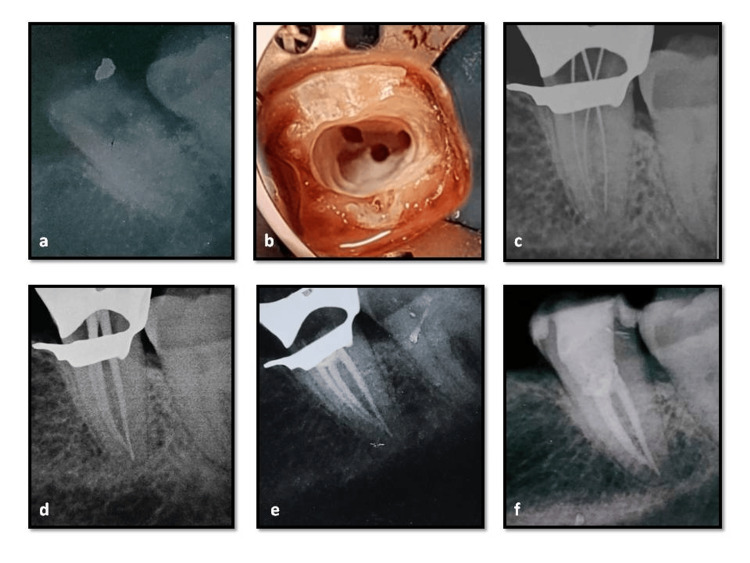
Case report-3 a) Preoperative radiograph; fused mesial and distal roots; b) access opening, three discrete canal orifices arranged in a C-shaped configuration; c) working length radiograph, narrow isthmus present between the mesial canals; d) master cone radiograph, all three canals merging at the apical third; e) obturation radiograph; f) postoperative radiograph.

Based on the preoperative radiographic assessment (Figure 5a), the appearance of fusion of mesial and distal roots led to a modified access cavity preparation. On examination after access opening, pulpal floor revealed three discrete canals orifices - Mesiobuccal, Mesiolingual, and Distal, arranged in a C-shaped configuration (Figure 5b). Canals were negotiated, and a working length radiograph showed all three canals merging at the apical third (Figure 5c). Chemo-mechanical preparation was completed with ProTaper Rotary System up to F2 in mesial canals and F3 in distal canals. The procedure was similar to the above-mentioned cases. In the next appointment, master cone selection was done (Figure 5d), and obturation was done using the single cone technique with AH plus sealer (Figure 5e), followed by composite restoration. Postoperative radiograph was taken (Figure 5f).

Case report four

**Figure 6:**
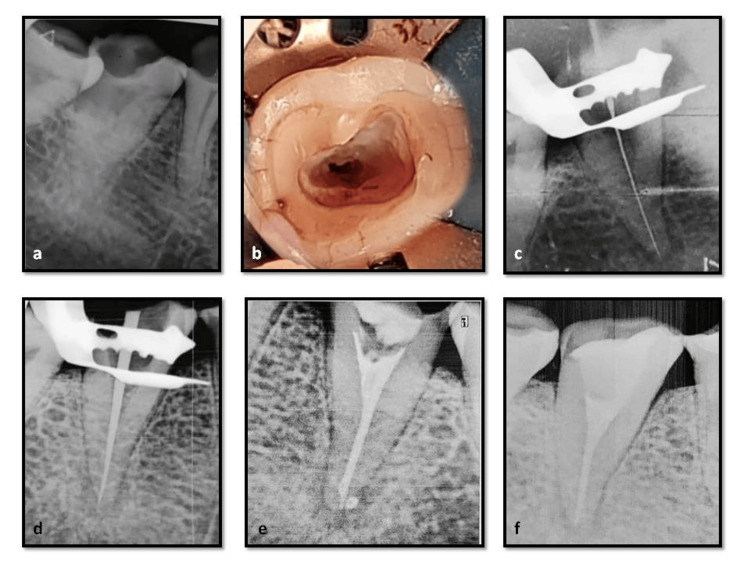
Case report-4 a) Preoperative radiograph, single conical root; b) access opening, single oval-shaped canal orifice; c) working length radiograph, single canal from orifice to the apex; d) master cone radiograph; e)obturation radiograph; f) postoperative radiograph.

As seen in the preoperative diagnostic radiograph (Figure 6a), a single oval-shaped orifice was located (Figure 6b). A single canal was negotiated from the orifice to the apex, and working length was determined (Figure 6c). Cleaning and shaping were done. Sectional obturation was done for the apical third, followed by thermoplasticized gutta-percha (Figures 6d, 6e). Postendodontic composite restoration was done (Figure 6f).

## Discussion

Recognizing the C-shaped configuration on the pulpal floor and determining the canal anatomy up to the root apex appeared to be a challenging task. Preoperative assessment can facilitate effective management. Certain radiographic findings, such as fused roots, a poorly defined pulp chamber floor, a working length radiograph with inserted instruments that appear to have a perforation in the furcation area, and instruments that tend to converge at the root apex, can lead a clinician to suspect C-shaped canal systems in second mandibular molars [11]. Although radiographs are quick, easy, and non-invasive, they might only provide a limited amount of information on their own. Understanding the anatomy is aided by cone-beam computed tomography (CBCT), magnifying loupes, microscopes, and a modified access cavity design [12].

C-shaped canals can have a variety of anatomical configurations over the length of the root. It can be difficult to clean and shape because of the presence of recesses, fins, anastomoses, and isthmuses. Soft tissue remnants and infectious debris may be retained in the fins or webs connecting the C-shaped root canals. For a satisfactory outcome, it is crucial to carry out an appropriate biomechanical preparation. C-shaped canals are traditionally cleaned and sculpted using hand and rotary files. Canal cross-sections that are roughly round are produced when both types of instruments are utilized, but up to 66% of the canal wall is left uninstrumented. These untreated areas of the root canal system could provide a sanctuary for bacteria. Tissue-dissolving irrigants and intracanal dressings must therefore be used in addition to instrumentation. It would be more successful to employ ultrasonics in conjunction with traditional therapy. In order to remove pulp tissue, circumferential filing must also be done carefully to prevent strip perforation [13].

Obturation of C-shaped canals may require certain modifications in the technique. The deeper penetration of the condensation instrument in several sites is required if the cold lateral condensation technique is used for obturation [14]. However, if lateral condensation is the only technique employed, sealing the isthmus becomes challenging. To ensure this, Barnett recommended that before seating the master cone in the mesial canal, a large diameter file be used in the most distal portion of the canal. After removing this file, the master cone is inserted in the distal canal, and accessory cones are then positioned in the center of the C-shaped canal [15]. The preferred method for the three-dimensional obturation of the C-shaped canals is warm vertical condensation. Applying thermoplasticized gutta-percha is more appropriate since this isthmus could not be sufficiently flared to allow for deeper spreader insertion [16].

Compaction can be enhanced when obturating a mandibular molar with a C-shaped canal by employing the EndoTec "zap and tap" approach. Before insertion, the EndoTec plugger (Dentsply, Milford, DE) must be preheated for four to five seconds. The hot instrument must then be moved in and out ten to fifteen times in quick, continuous strokes. After removing the plugger while it was still hot, a "cold spreader with insertion of additional accessory points" was utilized [17]. Walid also outlined a method for resolving the challenges while obturating. In order to downpack the major canals in a C-shaped canal, he recommended the use of two pluggers simultaneously [18].

The cases one and four, which represent the type I and type IV configurations, respectively, were managed with the thermoplasticized gutta-percha technique similar to that mentioned in the case report by Gade et al. [19]. Case two presents a semi-colon-shaped type II configuration in which obturation was done using the lateral condensation technique as reported in a case series by Jadhav et al. [20]. In case report three, there were three discrete canals orifices; thus, single cone obturation was done, similar to the one case reported by Zhou et al. [21]. Thus, all the cases were successfully treated with various obturation techniques, showing the diversity in endodontic management of C-shaped canals.

The most frequent cause of endodontic failure of C-shaped root canals was a leaky canal (45.2%) and isthmus (23.8%). Clinicians should be aware of the precise morphology of C-shaped root canals, try to remove the pulp tissue and microorganisms, and thoroughly seal the root canal system without any voids in order to avoid endodontic failure [22]. When treating such atypical root canal systems, the conventional treatment techniques must be modified.

Recommendations

Here is a summary of the recommendations for management of C-shaped canals:

Diagnostic and Anatomical Complexity

Due to the highly variable anatomy, conventional 2-dimensional periapical radiographs are often insufficient to accurately diagnose or visualize the full, complex three-dimensional nature of a C-shaped canal. Henceforth, Cone-Beam Computed Tomography (CBCT) is an essential tool for diagnosis and treatment planning.

Challenges in Treatment

Difficulty in debridement: The intricate fins, webs, and isthmuses are difficult to debride and clean using standard instrumentation alone. Failure to clean these areas is a primary cause of endodontic failure.

Difficulty in obturation: The irregular, ribbon-like, or C-shape of the canal system makes it very challenging to achieve a 3D obturation while using traditional techniques.

Recommendations for Successful Treatment

Enhanced visualization: Use of a Dental Operating Microscope (DOM) is essential for locating the complex C-shaped orifice.

Advanced irrigation: Copious irrigation with sodium hypochlorite (NaOCl) and EDTA, activated using sonics or ultrasonics, is essential to chemically dissolve tissue and clean the anatomical complexities that are difficult to reach with the instruments.

Specialized instrumentation: A combination of rotary and hand files, often used in a circumferential filing motion, is recommended to shape the canal walls as thoroughly as possible.

Advanced obturation: To properly fill the complex anatomy, warm vertical condensation or thermoplasticized gutta-percha techniques are recommended over cold lateral condensation.

## Conclusions

C‑shaped canals present significant diagnostic and treatment challenges due to their complex and variable anatomy. Conventional radiographs are often inadequate, making CBCT and the use of a dental operating microscope vital for accurate assessment and management. Successful treatment relies on enhanced irrigation, specialised instrumentation, and advanced obturation techniques to address the intricate canal morphology. By applying these strategies, clinicians can improve outcomes and reduce the risk of endodontic failure in these demanding cases.
